# Dietary Factors and Endometrial Cancer Risk: A Mendelian Randomization Study

**DOI:** 10.3390/nu15030603

**Published:** 2023-01-24

**Authors:** Xuemin Wang, Dylan M. Glubb, Tracy A. O’Mara

**Affiliations:** 1 Cancer Research Program, QIMR Berghofer Medical Research Institute, Brisbane, QLD 4006, Australia; 2 School of Biomedical Sciences, Faculty of Medicine, The University of Queensland, Brisbane, QLD 4072, Australia

**Keywords:** endometrial cancer, micronutrients, vitamins, minerals, protein, carbohydrate, fat, sugar, dietary patterns, Mendelian randomization

## Abstract

Given the strong association between obesity and endometrial cancer risk, dietary factors may play an important role in the development of this cancer. However, observational studies of micro- and macronutrients and their role in endometrial cancer risk have been inconsistent. Clarifying these relationships are important to develop nutritional recommendations for cancer prevention. We performed two-sample Mendelian randomization (MR) to investigate the effects of circulating levels of 15 micronutrients (vitamin A (retinol), folate, vitamin B6, vitamin B12, vitamin C, vitamin D, vitamin E, β-carotene, calcium, copper, iron, magnesium, phosphorus, selenium, and zinc) as well as corrected relative macronutrient intake (protein, carbohydrate, sugar and fat) on risks of endometrial cancer and its subtypes (endometrioid and non-endometrioid histologies). Genetically predicted vitamin C levels were found to be strongly associated with endometrial cancer risk. There was some evidence that genetically predicted relative intake of macronutrients (carbohydrate, sugar and fat) affects endometrial cancer risk. No other significant association were observed. Conclusions: In summary, these findings suggest that vitamin C and macronutrients influence endometrial cancer risk but further investigation is required.

## 1. Introduction

Endometrial cancer is the most commonly diagnosed invasive gynaecological cancer in developed countries, with 417,367 cases diagnosed in 2020 worldwide [1]. Cases are rising, with projections of a 40–50% increase in endometrial cancer incidence over the coming decade [2]. The most established risk factors for endometrial cancer are excessive exposure to estrogen and obesity [3]. Multiple studies have demonstrated robust and consistent associations between excess body mass index (BMI > 30 kg/m^2^) and increased endometrial cancer risk [3,4].

Given the strong relationship between obesity and endometrial cancer, it is thought that diet may play a role in the development of this cancer. Additionally, diet may mediate the role of endogenous estrogen, thereby promoting cancer growth [5]. However, the evidence for a role of nutrients in endometrial cancer risk has not been consistent [6]. A World Cancer Research Fund report found there was limited evidence available to assess the association between endometrial cancer and the dietary intake of most foods and thus no conclusions could be drawn [7]. Clarifying dietary relationships is important to make recommendations for endometrial cancer prevention.

Most studies of nutritional epidemiology rely on food frequency questionnaires to measure the consumption of foods and nutrients. This approach is prone to bias due to measurement error from participant self-reports [8]. Further, assessment of nutrient intake can be difficult due to fortified foods and the widespread consumption of vitamin supplements, resulting in inaccurate measurements [6]. Mendelian randomization (MR) is an approach that can be used to assess the relationship between a risk factor and disease using genetic variants as instrumental variables to proxy the risk factor of interest [9]. Because alleles are randomly assorted at conception, genetic variants are not influenced by confounding factors, such as measurement bias or reverse causation [10].

The aim of this study was to use MR analysis to investigate the relationship between essential dietary factors and endometrial cancer risk using genetically predicted relative macronutrient intake (fat, protein, sugar and carbohydrate)and genetically predicted circulating concentrations of 15 micronutrients (vitamins and minerals). Micronutrients selected are essential for biological processes related to cancer. Many have been previously assessed for association with endometrial cancer with inconsistent results reported [6,11].

## 2. Materials and Methods

### 2.1. Endometrial Cancer Data

Genome-wide association study (GWAS) summary statistics of endometrial cancer risk were from the latest Endometrial Cancer Association Consortium (ECAC) GWAS analysis (12,906 cases and 108,979 controls) [12]. To avoid bias due to overlapping sample sets in MR analyses, UK Biobank samples were removed from the ECAC summary statistics, resulting in 12,270 endometrial cancer cases and 46,126 controls [13]. In a secondary analysis, we investigated relationships between the circulating micronutrients and macronutrient dietary compositions and endometrial cancer subtypes using ECAC GWAS results restricted to cases with either endometrioid histology (8758 cases), or non-endometrioid histology (1230 cases). Histological subtypes of endometrial cancer were confirmed based on pathology reports, and detailed study descriptions have previously been reported [12,14].

### 2.2. Relative Intake of Macronutrients (Dietary Composition) Data

Instrument variables for four macronutrient dietary compositions (relative intake of carbohydrate, sugar, protein, and fat) were extracted from Meddens, et al. [15]. GWAS discovery of the four dietary compositions were performed by the Social Science Genetic Association Consortium and included participants of mainly European ancestry, aged 27–71 years. Self-report questionnaires containing questions on more than 70 food items were used to estimate the relative composition of macronutrients. Analyses of relative intake of carbohydrate, protein and fat included 268,922 participants, while 235,391 participants were included in analysis of relative intake of sugar. Macronutrient intake is expressed as % of total energy intake (E%). Full details of the study are provided by Meddens, et al. [15].

### 2.3. Micronutrients Data

We conducted a search of published GWAS performed among individuals of European ancestry for circulating concentrations of minerals and vitamins in the GWAS catalog and PubMed (last search performed in 31 May 2022). A total of 15 micronutrients vitamin A (retinol) [16], folate [17], vitamin B6 [18], vitamin B12 [17], vitamin C [19], vitamin D [20], vitamin E [21], β-carotene [22], calcium [23], copper [24], iron [25], magnesium [26], phosphorus [27], selenium [28], and zinc [29] were included in the Mendelian analysis. Although vitamin C, vitamin D and selenium have previously been investigated for their role in endometrial cancer risk by MR [20,30,31], we repeated this analysis due to the slightly different endometrial cancer GWAS dataset used in the current study due to the removal of the UK Biobank strata. Genetic variant estimates are reported as the association with a one standard deviation (SD) change in micronutrient levels.

### 2.4. Mendelian Randomization Analysis

This study referred to the Strengthening the Reporting of Observational Studies in Epidemiology using Mendelian Randomization (STROBE-MR) guidelines [32]. Three core assumptions are required to be met in order for MR analysis to yield valid causal estimates between exposures of interest and disease outcomes: (1) genetic variants are robustly associated with exposure of interest; (2) genetic variants are not associated with any known or potential confounders; and (3) genetic variants are not associated with the outcome (i.e., endometrial cancer risk) through any other path than through the exposure [33]. We used two-sample MR analysis framework, in which GWAS summary statistics from different studies of exposure and outcome are used to estimate causal effects and increase the statistical power and precision of the MR analysis.

We selected genetic variants that were reliably (*p* < 5 × 10^−8^) and independently (linkage disequilibrium; LD r^2^ ≤ 0.001 within a window of 10 Mb) associated with each nutrient as instrumental variables. LD was estimated using the 1000 Genomes EUR reference panel. We also removed palindromic variants (those with A/T or G/C alleles) with intermediate allele frequencies (allele frequency between 0.42 and 0.58) to prevent strand ambiguity errors. The variance explained by the selected instrumental variables for each nutrient and F-statistics for instrument variable strength were estimated as per Yarmolinsky, et al. [34]. An F-statistic > 10 was used to indicate sufficient instrument strength. Table 1 and Table S1 provide detailed information for macro- and micronutrient instrumental variables. Power for Mendelian randomization analyses to detect an association between a one SD change in genetically predicted relative intake of macronutrients or levels of micronutrients and endometrial cancer risk were calculated for cut-offs reflecting weak, moderate and strong effects (i.e., OR > 1.1, OR > 1.2, and OR > 1.4) using the mRnd (https://shiny.cnsgenomics.com/mRnd/ (accessed on 11 January 2023)) online MR power calculator [35].

Mendelian randomization analyses were performed using the TwoSampleMR R package (version 0.5.5) [36]. The inverse-variance weighted (IVW) approach was used as the primary analysis to estimate the association between each macro- or micronutrient and endometrial cancer risk, apart from traits with a single instrumental variable (i.e., vitamin B6 and β-carotene) where the Wald ratio was used to estimate effects. For palindromic variants, we used allele frequencies to infer positive strand alleles by setting the level of strictness to action = 2 in the harmonise_data function. The IVW method provides reliable estimates when all instruments are valid, meeting the three core MR assumptions as provided above. If instruments affect the outcome other than through the exposure of interest (i.e., assumptions 2 and 3, “horizontal pleiotropy”), the IVW approach may still provide reliable estimates if the net effect of these other pathways on the outcome is zero. However, where the net effects is not zero, bias is introduced from “unbalanced horizontal pleiotropy”. The direction of bias depends on whether the overall effect of the invalid instruments increases or decreases the risk of the outcome.

To investigate possible bias from invalid variants and possible violations of assumptions 2 and 3, when genetic instruments contained at least three variants, we performed three sensitivity analyses using alternative MR methods: MR-Egger regression, weighted median, and the MR-pleiotropy residual sum and outlier (MR-PRESSO) approaches. Each of these MR methods employ a unique set of assumptions and can provide reliable estimates in the presence of invalid variants at the expense of power. MR-Egger regression provides a formal statistical test of the presence of such unbalanced horizontal pleiotropy and can be reliable in the presence of such bias [37], albeit at the cost of low power and precision. However, estimates from MR-Egger are at risk of bias if the genetic instrument contains outlying variants or if horizontal pleiotropic effects are correlated with the exposure. In contrast, the weighted median method is reliable in the presence of outlying instruments assuming less than half of the variants contributing the genetic instrument are invalid [38]. The MR-PRESSO approach identifies and removes outlying variants and can then provide unbiased estimates, assuming the remaining variants contributing the genetic instrument are valid [39].

As a further means of detecting violations in the assumptions underlying MR, we tested for the presence of heterogeneity in the causal effect estimates across the individual variants. For this purpose, we calculated Cochran’s Q [40] with its associated *p*-value and visually inspected a funnel plot (i.e., where variant-specific associations were plotted against their inverse standard errors) to check if the causal estimates from weaker variants were distributed in a particular direction. We also assessed leave-one-out plots for IVW estimates to ensure estimates were not susceptible to highly influential genetic variants in the model.

Exposures with MR analysis results that were below a Bonferroni correction, accounting for multiple testing (*p* < 0.0026; i.e., 0.05/19 tested exposures), were classed as having strong evidence for association with endometrial cancer. Exposures with MR results with *p* ≥ 0.0026 and <0.05 were classed as having suggestive evidence for an association with endometrial cancer.

## 3. Results

Of the dietary factors studied, only vitamin C was found to have strong evidence for an association with endometrial cancer risk, with estimates (OR = 1.41; 95% CI 1.16–1.72; *p* = 7 × 10^−4^; Table 2, Table S2) similar to that previously reported [30]. In secondary analysis, assessing risk by endometrial cancer histological subtypes, strong evidence of a protective effect for relative intake of sugar was observed for non-endometrioid endometrial cancer (OR = 0.08; 95% CI 0.02–0.34; *p* = 6 × 10^−4^). There was suggestive evidence that relative intake of carbohydrate (*p* = 0.03) and sugar (*p* = 0.009) reduced risk of endometrial cancer, and that relative intake of fat (*p* = 0.01) increased risk of endometrial cancer. Secondary analysis also found suggestive evidence for an association between copper levels and increased endometrioid endometrial cancer risk (*p* = 0.04). Results from power calculations for MR IVW analyses are presented in Table S3.

**Figure 1:**
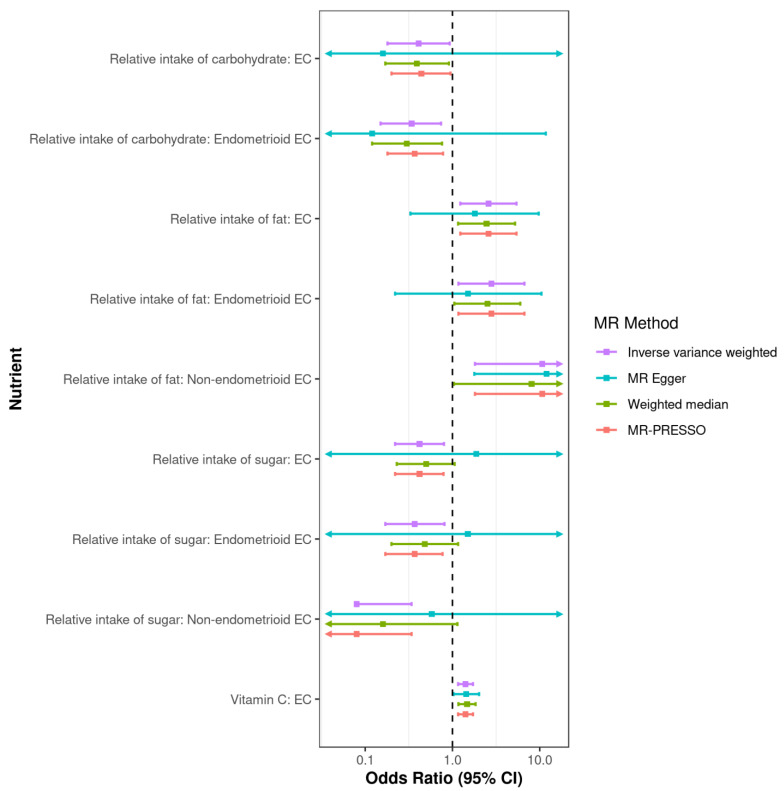
Plot of MR sensitivity analyses for dietary that were associated with endometrial cancer (EC) risk by the primary IVW analysis.

Exposures that exhibited at least suggestive evidence of an association with endometrial cancer were further scrutinized by sensitivity analyses (Figure 1, Table S2). Estimates were consistent across all sensitivity analyses, apart from the association between relative intake of sugar with endometrial cancer (of all histological subtypes) and endometrioid endometrial cancer risk by MR-Egger regression. However, the statistical power of MR-Egger regression is known to be very low, which is reflected in the wide confidence intervals for the estimate by this approach. Further, there was no evidence of pleiotropy detected by the MR Egger intercept (P_intercept_ > 0.05) and no significant heterogeneity observed across the genetic instruments (Cochrane’s Q *p*-value > 0.05; Table S2). The genetic instrument for copper only contained two variants and could not be assessed by the MR sensitivity analyses. Visual inspection of the IVW forest plot did not find that the causal estimate was unduly influenced by the result of one variant, with consistent effects on endometrioid endometrial cancer risk observed for both variants.

## 4. Discussion

We performed MR analyses using large scale GWAS summary statistics and found some evidence for an association of endometrial cancer risk with genetically predicted vitamin C levels and macronutrient dietary patterns. Specifically, we found suggestive evidence that higher relative intake of fat may increase endometrial cancer risk while higher relative intake of carbohydrate or sugar may reduce risk. Apart from vitamin C, there was little evidence that genetically predicted levels of micronutrients (vitamins and minerals) affect endometrial cancer risk.

Increased BMI and waist circumference are both strongly associated with endometrial cancer risk, with associations reported by observational and MR analyses [3,4,12]. Thus, the results for macronutrient dietary patterns and endometrial cancer risk are consistent with a prior MR study that reported a lower relative intake of carbohydrate and a higher relative intake of fat are associated with increased BMI and waist circumference [41]. Observational studies assessing the relationship between dietary patterns and endometrial cancer risk have been inconsistent, this could be due to many factors, including confounding introduced by self-reported food questionnaires, recall bias and, in relation to case-control studies, to reverse causation [6,11]. However, our results are consistent with a large prospective study which reported a high carbohydrate diet is protective for endometrial cancer development [42]. Similar to our results, this study also found high total sugar intake was protective for endometrial cancer. Our results are also consistent with a study assessing dietary fat in a case-control analysis, with high energy percentage from fat observed to increase risk of endometrial cancer [43]. A consistent direction of effect was seen for risk estimates for relative intake of carbohydrate, fat and sugar across endometrial cancer and its histological subtypes (endometrioid and non-endometrioid). Although the magnitude of the estimates were higher for non-endometrioid histologies, the confidence intervals were wide, perhaps due to the smaller numbers in this subgroup.

A clinical trial including endometrial cancer patients reported that a ketogenic diet (70% fat, 25% protein and 5% carbohydrate, no calorie restriction implemented) was found to have beneficial effects for a range of features, which included reduced central obesity and serum insulin, and increased physical function [44,45]. However, the effect of this diet on endometrial cancer risk has not been assessed. Our results suggest that a ketogenic diet may not be effective in reducing endometrial cancer risk.

We found higher genetically predicted vitamin C was associated with increased endometrial cancer risk, consistent with a recently published MR study [30]. Observational studies of vitamin C intake for endometrial cancer have yielded conflicting results. This could be due to study design (cohort vs. case-control study), consideration for confounding and measurement of vitamin C intake (e.g., food intake alone vs. supplementation). A meta-analysis of nine studies assessing vitamin C intake from food reported a protective effect of increased vitamin C intake on endometrial cancer risk [46]. Given these results were based on food intake questionnaires, it is difficult to disentangle the effects of other components of vitamin C rich food. The current MR study has allowed for assessment of genetically predicted vitamin C levels from plasma. The finding that increased vitamin C levels may increase endometrial cancer risk suggests that supplementation of vitamin C in high risk populations should be avoided. Further analysis is required to make clinical recommendations and to determine a potential casual mechanism.

Apart from vitamin C, genetically predicted copper levels were the only other micronutrient factor observed to be associated with endometrial cancer risk, specifically, increased risk of endometrioid endometrial cancer. However, it is difficult to draw conclusions from this result given that the association was suggestive (P_IVW_ = 0.04) and sensitivity analyses could not be performed because only two variants were included in the genetic instrument. Thus, larger GWAS for copper levels are required to better define the genetic instrument for MR analyses and clarify its relationship with endometrial cancer risk.

There are a number of limitations for this study, including the fact that all analyses were performed using European participants only and, thus, it is difficult to assess whether these results can be extrapolated to other populations. Despite using the largest GWAS datasets available to extract genetic variants, only a small amount of trait variance could be explained by instrumental variables, which has resulted in limited statistical power to detect weak to moderate associations for many dietary factors. Therefore, these analyses should be repeated as larger GWAS become available. GWAS for circulating vitamin E and retinol concentrations were adjusted for BMI [16,21], which may cause collider bias in MR estimates [47]. Because we did not consider macronutrient by type we could not assess whether there were differences between the causal effects of certain subtypes or macronutrient (e.g., type of fat or carbohydrate) and overall macronutrient type. By using relative macronutrient intake, we could not assess macronutrients independently and we were also unable to explore total energy consumption. Future studies to examine this could be warranted as we note in a small case-control analysis, total energy intake was reported to associate with increased endometrial cancer risk, but there were no associations found for any individual nutrients [48].

This study suggests that altering dietary patterns to have a macronutrient composition of lower fat and higher carbohydrate or sugar intake could result in lowering endometrial cancer risk in the general population. We also found increased serum levels of vitamin C associate with increased risk of endometrial cancer, consistent with a previous MR study [30]. However, further study is required to explore these relationships before dietary recommendations could be made.

## Figures and Tables

**Table 1:** Details of genetic instruments for investigated dietary factors.

Exposure	Reference	Sample Size	Number of IVs	R^2^	F-Statistic	Consortia
*Macronutrients*						
Relative intake of carbohydrates	[5]	268,922	12	0.18%	39.5	UKBB, Netherlands (Lifelines, RSI/II/III), UK (ALSPAC, Fenland), USA (FHS, HRS, WHI-GARNET, WHI-HIPFX, WHIMS+), EPIC-InterAct, and DietGen
Relative intake of fat	[5]	268,922	6	0.13%	58.8	
Relative intake of protein	[5]	268,922	7	0.14%	53.7	
Relative intake of sugar	[5]	235,391	9	0.19%	49.8	UKBB, Netherlands (Lifelines, RSI/II/III), UK (ALSPAC, Fenland), USA (FHS, HRS, WHI-GARNET, WHI-HIPFX, WHIMS+), and EPIC-InterAct
*Micronutrients: Vitamins*						
Vitamin A (retinol) ^1^	[17]	8902	2	0.63%	28.4	ATBC, PLCO, NHS-CHD, NHS-T2D, NHS-CGEMS, InCHIANTI
B vitamin: folate	[18]	37,341	2	0.76%	142.6	Icelandic, Danish-Inter99, Danish-Health2006
B vitamin: vitamin B12	[18]	45,576	11	5.13%	224	
B vitamin: vitamin B6	[19]	4763	1	1.02%	49	NHS-CGEMS, FHS-SHARe
Vitamin C	[20]	52,018	11	1.79%	86	Fenland, EPIC-Norfolk, InterAct, EPIC-CVD
Vitamin D	[21]	438,870	76	3.68%	201.8	UKBB
Vitamin E ^1^	[22]	8781	3	0.39%	11.4	ATBC, PLCO, and NHS
β-carotene	[23]	3881	1	2.48%	98.6	InCHIANTI, WHAS I and WHAS II, and ATBC
*Micronutrients: Minerals*						
Calcium	[24]	61,079	7	0.84%	74	AGES, ARIC, BLSA, CHS, CoLaus, CROATIA-Korcula, CROATIA-Split, CROATIA-Vis, FHS, HABC, InCHIANTI, LBC1936, LOLIPOP EW A, LOLIPOP EW P, LOLIPOP EW610, OGP Talana, ORCADES, RS, SHIP, BRIGHT, Bus Santé, INGI-Carlantino, INGI-FVG, INGI-CILENTO, KORA-F3, KORA-F4, LURIC, PIVUS, SHIP-Trend, TwinsUK
Copper	[25]	5594	2	1.94%	55.4	EIPC-Potsdam, PIVUS, QIMR
Iron	[27]	246,139	14	2.63%	314.9	deCODE genetics, INTERVAL study, Danish Blood Donor Study
Magnesium	[28]	23,829	6	1.45%	58.5	ARIC, FHS, RS
Phosphorus	[29]	16,264	5	0.75%	41.1	CHS, FHS, ARIC, RS, KORA-F3, KORA-F4, Health ABC, CROATIA-Vis
Selenium	[30]	9639	2	2.12%	104.3	CARDIA, JoCo, NHS, HPFS, QIMR, and ALSPAC
Zinc	[31]	2603	2	4.59%	62.6	QIMR and ALSPAC

ALSPAC—the Avon Longitudinal Study of Parents and Children; ARIC—Atherosclerosis Risk in Communities; ATBC—the Alpha-Tocopherol, β-Carotene Cancer Prevention Study; BLSA—Baltimore Longitudinal Study of Aging; CARDIA—Coronary Artery Risk Development in Young Adults; CHS—Cardiovascular Healthy Study; CoLaus—The CoLaus cohort; DietGen—DietGen Consortium; EPIC—The European Prospective Investigation into Cancer and Nutrition study; FHS—Framingham Heart Study; HPFS—the Health Projessionals Follow-up Study; HRS—Health and Retirement Study; InCHIANTI—a population-based study of the older population living in the Chianti region of Tuscany, Italy; JoCo—Johnston County Osteoarthritis Project; Lifelines—the Lifelines Cohort Study; LOLIPOP—London Life Sciences Population study; NHS—the Nurses’ Health Study; NHS-CGEMS—the Nurses’ Health Study of Cancer Genetic Markers of Susceptibility; NHS-CHD—the Nurses’ Health Study of coronary heart disease; NHS-T2D—the Nurses’ Health Study of type 2 diabetes; PLCO—the Prostate, Lung, Colorectal and Ovarian Cancer Screening Trial Study; RS—the Rotterdam Study; RSI—Rotterdam Study I; RSII—Rotterdam Study II; RSIII—Rotterdam Study III; UKBB—UK Biobank; WHAS—the Women’s Health and Aging Study; WHI-GARNET—Women’s Health Initiative—Genomics and Randomized Trials Network; WHI-HIPFX—Women’s Health Initiative—Hip Fracture GWAS; WHIMS+—Women’s Health Initiative—Memory Study.

**Table 2:** Inverse variance weighted Mendelian randomization results for dietary factors by endometrial cancer histological subtype.

Exposure	Number of IVs	EC (All Histological Subtypes)		Endometrioid EC		Non-Endometrioid EC	
		OR (95% CI)	*p*-Value	OR (95% CI)	*p*-Value	OR (95% CI)	*p*-Value
*Macronutrients*							
Relative intake of carbohydrate	12	**0.41 (0.18, 0.93)**	**0.03**	**0.34 (0.15, 0.74)**	**0.006**	0.25 (0.04, 1.57)	0.14
Relative intake of fat	6	**2.59 (1.23, 5.42)**	**0.01**	**2.8 (1.17, 6.68)**	**0.02**	**10.69 (1.81, 63.1)**	**0.009**
Relative intake of protein	7	1.26 (0.47, 3.38)	0.64	1.4 (0.47, 4.14)	0.55	3.5 (0.43, 28.48)	0.24
Relative intake of sugar	9	**0.42 (0.22, 0.8)**	**0.009**	**0.37 (0.17, 0.81)**	**0.01**	***0.08 (0.02, 0.34)***	***6 × 10^−4^***
*Micronutrients: Vitamins*							
Vitamin A (retinol)	2	0.63 (0.16, 2.39)	0.49	0.63 (0.11, 3.67)	0.61	4.98 (0.23, 109.47)	0.31
B vitamin: Folate	2	1.11 (0.85, 1.44)	0.43	1.12 (0.8, 1.57)	0.50	1.31 (0.63, 2.71)	0.47
B vitamin: Vitamin B12	10	1.03 (0.93, 1.13)	0.58	1 (0.9, 1.12)	0.99	0.99 (0.72, 1.36)	0.94
B vitamin: Vitamin B6 ^1^	1	0.93 (0.71, 1.22)	0.60	0.95 (0.7, 1.3)	0.76	0.88 (0.42, 1.85)	0.73
Vitamin C	11	***1.41 (1.16, 1.72)***	***7 × 10^−4^***	1.32 (0.96, 1.83)	0.09	1.39 (0.87, 2.22)	0.16
Vitamin D	75	0.93 (0.8, 1.09)	0.40	0.92 (0.79, 1.08)	0.32	1.01 (0.73, 1.41)	0.94
Vitamin E	3	1.27 (0.62, 2.61)	0.51	1.66 (0.58, 4.75)	0.35	0.9 (0.12, 6.89)	0.92
β-carotene ^1^	1	1.04 (0.85, 1.29)	0.68	0.97 (0.77, 1.23)	0.79	1.63 (0.92, 2.87)	0.09
*Micronutrients: Minerals*							
Calcium	7	0.96 (0.55, 1.66)	0.87	1.06 (0.61, 1.86)	0.83	1.29 (0.33, 5.11)	0.72
Copper	2	1.11 (0.92, 1.34)	0.27	**1.17 (1.01, 1.35)**	**0.04**	0.9 (0.63, 1.28)	0.55
Iron	14	1.1 (0.9, 1.33)	0.35	1.07 (0.85, 1.34)	0.59	1.2 (0.87, 1.65)	0.26
Magnesium	6	0.21 (0.02, 2.69)	0.23	0.11 (0.01, 1.96)	0.13	0.31 (0, 85.93)	0.68
Phosphorus	5	1.25 (0.83, 1.88)	0.29	1.35 (0.85, 2.15)	0.21	0.93 (0.3, 2.92)	0.90
Selenium	2	1.03 (0.77, 1.38)	0.84	1.08 (0.85, 1.38)	0.52	1.08 (0.46, 2.51)	0.86
Zinc	2	0.89 (0.78, 1.02)	0.09	0.89 (0.74, 1.08)	0.24	0.89 (0.66, 1.2)	0.44

Abbreviations—IV: instrumental variable; EC: endometrial cancer; OR: odds ratio; CI: confidence interval. Results with a nominally significant result (*p* < 0.05) are bolded, results passing Bonferroni correction for multiple testing are italicized and bolded. ^1^ This was analyzed by Wald Ratio as there was only one IV for this nutrient.

## Data Availability

All data analyzed are publicly available from the relevant study groups.

## Supplementary Materials

The following supporting information can be downloaded at: https://www.mdpi.com/article/10.3390/nu15030603/s1, Table S1: Details of variants included in genetic instruments; Table S2: Results for Mendelian randomization analyses; Table S3: Estimation of power for Mendelian randomization analyses for endometrial cancer risk based on total sample size and proportion of phenotypic variance of macro- and micronutrients explained by instruments.
